# Extensive weight loss reduces glycan age by altering IgG N-glycosylation

**DOI:** 10.1038/s41366-021-00816-3

**Published:** 2021-05-03

**Authors:** Valentina L. Greto, Ana Cvetko, Tamara Štambuk, Niall J. Dempster, Domagoj Kifer, Helena Deriš, Ana Cindrić, Frano Vučković, Mario Falchi, Richard S. Gillies, Jeremy W. Tomlinson, Olga Gornik, Bruno Sgromo, Tim D. Spector, Cristina Menni, Alessandra Geremia, Carolina V. Arancibia-Cárcamo, Gordan Lauc

**Affiliations:** 1 Translational Gastroenterology Unit and NIHR Oxford Biomedical Research Centre, Nuffield Department of Medicine, University of Oxford, Oxford, UK; 2 Faculty of Pharmacy and Biochemistry, University of Zagreb, Zagreb, Croatia; 3 Genos Glycoscience Research Laboratory, Zagreb, Croatia; 4 Oxford Centre for Diabetes and NIHR Oxford Biomedical Research Centre, Endocrinology and Metabolism, Radcliffe Department of Medicine, University of Oxford, Oxford, UK; 5 The Department of Twin Research, King’s College London, St Thomas’ Hospital, London, UK; 6 Department of Upper GI Surgery, Oxford University Hospitals, Oxford, UK

**Keywords:** Glycoconjugates, Immunology, Obesity

## Abstract

**Background:**

Obesity, a major global health problem, is associated with increased cardiometabolic morbidity and mortality. Protein glycosylation is a frequent posttranslational modification, highly responsive to inflammation and ageing. The prospect of biological age reduction, by changing glycosylation patterns through metabolic intervention, opens many possibilities. We have investigated whether weight loss interventions affect inflammation- and ageing-associated IgG glycosylation changes, in a longitudinal cohort of bariatric surgery patients. To support potential findings, BMI-related glycosylation changes were monitored in a longitudinal twins cohort.

**Methods:**

IgG N-glycans were chromatographically profiled in 37 obese patients, subjected to low-calorie diet, followed by bariatric surgery, across multiple timepoints. Similarly, plasma-derived IgG N-glycan traits were longitudinally monitored in 1680 participants from the TwinsUK cohort.

**Results:**

Low-calorie diet induced a marked decrease in the levels of IgG N-glycans with bisecting GlcNAc, whose higher levels are usually associated with ageing and inflammatory conditions. Bariatric surgery resulted in extensive alterations of the IgG N-glycome that accompanied progressive weight loss during 1-year follow-up. We observed a significant increase in digalactosylated and sialylated glycans, and a substantial decrease in agalactosylated and core fucosylated IgG N-glycans (adjusted *p* value range 7.38 × 10^−04^–3.94 × 10^−02^). This IgG N-glycan profile is known to be associated with a younger biological age and reflects an enhanced anti-inflammatory IgG potential. Loss of BMI over a 20 year period in the TwinsUK cohort validated a weight loss-associated agalactosylation decrease (adjusted *p* value 1.79 × 10^−02^) and an increase in digalactosylation (adjusted *p* value 5.85 × 10^−06^).

**Conclusions:**

Altogether, these findings highlight that weight loss substantially affects IgG N-glycosylation, resulting in reduced glycan and biological age.

## Highlights

- Obesity is associated with inflammation-related agalactosylated and bisected IgG glycoforms.
- IgG bisected glycoforms decrease after low-calorie diet.
- IgG galactosylation and sialylation increase after bariatric surgery-induced weight loss.
- Progressive decrease of BMI is associated with increased IgG galactosylation, implying a reduction of biological age.

## Introduction

The global prevalence of obesity has risen dramatically in the past decades, and it is now a pandemic [1]. According to the World Health Organisation, over 650 million individuals are obese, accounting for 13% of the world’s adult population. Obesity confers risk for metabolic syndrome, contributing to type 2 diabetes and cardiovascular disease (CVD) development [2]. Metabolic syndrome is linked to a chronic systemic and tissue low-grade inflammation. This long-term inflammatory state, known as inflammageing, accelerates the biological ageing process and exposes the organism to pathologies by weakening the immune system [3, 4]. Obesity-related inflammageing results in impaired innate and adaptive immune function, and it is characterised by high serum levels of IL-6, TNF-α and CRP [5]. Altered protein N-glycosylation has been found to be one of the hallmarks of inflammageing [4, 6]. The human circulating N-glycome represents the entire set of glycans that are covalently attached to plasma proteins through nitrogen on an asparagine residue. N-glycans are essential for life and are involved in many physiological processes [7], including receptor regulation and cell adhesion, signal transduction, protein trafficking and folding. Glycosylation has a fundamental role in the innate and adaptive immune responses, accentuated by the fact that all five classes of immunoglobulins (Ig) bear N-glycans. In this regard, IgG is probably the most investigated glycoprotein, whose effector functions are controlled by its Fc-bound glycans [8].

Inter-individual differences in the pace of biological ageing is an intriguing concept that may explain why some people stay healthy until their late chronological age, while others age faster and have shorter life expectancy. Progressive age-related changes of IgG N-glycosylation have been extensively studied [7, 9, 10] and the GlycanAge model has been proposed to express the difference between chronological and IgG N-glycome ageing [11]. The age of the IgG N-glycome can be estimated through the levels of agalactosylated species, which increase with ageing and are associated with enhanced immune activation [12]. The opposite applies for digalactosylated IgG glycoforms, which are usually related to a younger age. In addition to age-related changes, specific IgG glycosylation patterns have already been associated with CVD risk score and subclinical atherosclerosis in two large independent UK cohorts [13]. Moreover, proinflammatory IgG N-glycome has been associated with hypertension [14–16], and similar IgG glycosylation patterns were associated with increased body mass index (BMI) and measures of central obesity [17, 18].

Studies in mouse models further corroborate the importance of differential IgG glycoforms in CVD pathogenesis. It has been shown that hyposialylated IgG (corresponding to an old-like IgG N-glycome) can induce obesity-related hypertension and insulin resistance in B-cell-deficient mice, through activation of the endothelial FcγRIIB [19, 20]. These findings indicate that the IgG N-glycome could represent more than a biomarker of inflammation and ageing, since distinctive IgG glycoforms act as effector molecules in certain pathologies. IgG agalactosylation signatures have also been associated with inflammatory bowel diseases (IBD) and their severity [21]. Altered N-glycosylation patterns were not only reported on IgG N-glycome but also in T cells [22]. Glycosyltransferases, the specific enzymes involved in N-glycosylation play a pivotal role in generating aberrant N-glycoprofiles. Data from two independent European IBD cohorts with ulcerative colitis (UC) have shown that variants on the MGAT5 gene, coding for N-acetylglucosaminyltransferase [GnT]-V enzyme affect the glycosylation pattern in T cells and IgG [23]. Intronic SNPs causing MGAT5 downregulation impact the glycosylation pattern, resulting in T-cell hyperactivation. These alterations correlated with UC pathogenesis and severity of the disease and show the possible use of IgG glycome signatures for patient stratification.

In obesity, supplementation with a precursor of sialic acid protects obese mice from hypertension and insulin resistance induction by reverting an old age-associated IgG N-glycome into a young age-associated IgG N-glycome [20, 24]. However, studies exploring the possibilities of converting an old age- into a young age-associated IgG N-glycome by metabolic intervention in humans are limited. Of note, only one small study indicated that high-intensity interval training can rejuvenate the IgG N-glycome [25].

Bariatric surgery is very effective for the treatment of severe obesity [26]. The resulting weight loss impacts energy balance and metabolism, contributing to the increased insulin response, improved glycaemic control and reduction of total body fat, leading to decreased CVD risk and mortality [27].

In this study we aimed to determine whether weight loss modifies glycan age related to inflammation and ageing, in a longitudinally monitored cohort of obese individuals undergoing low-calorie diet and then bariatric surgical interventions. We also investigated BMI-related N-glycosylation changes in the longitudinal TwinsUK cohort, the largest cohort of adult twins with the most detailed clinical database in the world.

## Methods

### Study populations

#### Bariatric cohort

This exploratory cohort included 37 participants, recruited at Oxford University Hospitals to the Gastrointestinal Illnesses study (Ref: 16/YH/0247). All patients were characterised by metabolic status and medical history. Bariatric patients were considered eligible in accordance with National Institute for Health and Care Excellence (NICE) and local guidelines.

Patients with a history of alcoholism and/or ongoing anticoagulant treatment were excluded. Patients were also excluded in case of pregnancy, active substance abuse or uncontrolled psychiatric condition including eating disorders. Participants were sampled at baseline and subjected to 3-week low calorie carbohydrate-restricted diet (900 kcal/day, maximum 100 g of carbohydrates per day), followed by bariatric surgery. The sequential follow-up timepoints included the day of the surgery (baseline), at 20% of weight loss after 6.54 ± 3.4 months (mean ± IQR) and 12.47 ± 6.55 months post-op. Characteristics of the bariatric cohort are shown in Table 1.

**Table 1:** Demographic characteristics of the bariatric cohort.

Characteristics	Bariatric cohort				
Total No. of participants (*N*)	37				
No. of participants Sleeve Gastrectomy (SG), *N* (%)	25 (68%)				
No. Roux-en-Y Gastric Bypass RYGB *N* (%)	12 (32%)				
No. of participants each timepoint (*N*)	Pre-op low-calorie diet		Time of surgery	1st post-op timepoint	2nd post-op timepoint
	8		37	30	24
Age of participants, mean ± SD, years	Before diet	End of diet	Time of surgery	1st post-op timepoint	2nd post-op timepoint
	46.5 ± 9.27	46.5 ± 9.27	48.15 ± 9.34	48.41 ± 8.91	49 ± 9.34
BMI^a^ each timepoint, mean ± SD, kg/m2	Before diet	End of diet	Time of surgery	1st post-op timepoint	2nd post-op timepoint
	48.53 ± 4.13	46.60 ± 4.21	46.21 ± 4.75	36.01 ± 5.07	32.82 ± 5.17
Female, sex, *N* (%)	33 (89%)				
Type 2 diabetes, *N* (%)	6 (16%)				

^a^BMI reference values: <18.5 (underweight), 18.5–24.9 (normal weight), 25–29.9 (overweight), >30 (obese).

#### TwinsUK cohort

We have analysed a total of 6032 plasma samples from 2146 participants of the TwinsUK study, collected at multiple timepoints over a 20 year-period, as a replication cohort [28]. These included 1865 individuals sampled at three timepoints, 156 individuals sampled at two timepoints and 125 individuals sampled only once. Following the plasma N-glycome analysis, glycan data underwent quality control (see Statistical analysis section), which decreased the dataset to 5889 samples (measurements). Out of these 5889 measurements, we have proceeded with statistical analysis on a subset of 3742 samples (measurements) that had information on BMI available. Description of the TwinsUK cohort is provided in Table 2.

**Table 2:** Demographic characteristics of the TwinsUK cohort.

Characteristics	TwinsUK cohort (BMI subset)
No. of participants (*N*)	1680
No. of glycan measurements (*N*)	3742
Baseline age, mean ± SD, years	53.23 ± 10.86
Follow up time, mean ± SD, years	7.90 ± 5.66
Female sex, *N* (%)	1680 (100)
Baseline BMI, mean ± SD, kg/m^2^	25.45 ± 4.53

### N-glycome analysis

#### Experimental design and method performance

All samples were transferred to their designated positions on a 96-well plate according to predetermined experimental design, that was blocked on case–control status, sex and age. Standard and blank samples were included in every batch (96-well plate) for quality control and batch correction.

The sensitivity of the method for IgG N-glycome profiling was previously determined [29] based on the minimal starting amount of IgG (µg) as well as on the proportion of its starting amount which is finally analysed chromatographically (i.e. applied to the column). Namely, the minimal starting amount of IgG is 10 µg, i.e. the minimal amount of IgG required for the reliable quantification of its released N-glycans using fluorescence detection is 0.42 µg. The precision of the method is reported with coefficients of variation (CV, %) that are calculated from the relative abundance of each glycan peak (%) of standard samples. Herein, five standard samples per plate were analysed, giving the average CV value for directly measured IgG glycan peaks of 4.28% (range 0.44–15.65%), whereas calculated derived glycan traits gave the average CV value of 1.63% (range 0.17–4.39%).

#### Isolation of IgG from human plasma

IgG was isolated from plasma samples by affinity chromatography as described previously [30]. In brief, IgG was isolated in a high-throughput manner, using 96-well protein G monolithic plates (BIA Separations, Slovenia), starting from 100 μl of plasma. Plasma was diluted 7× with phosphate buffered saline (PBS; Merck, Germany) and applied to the protein G plate. IgG was eluted with 1 ml of 0.1 M formic acid (Merck, Germany) and immediately neutralised with 1 M ammonium bicarbonate (Acros Organics, USA).

#### N-glycan release from IgG and total plasma proteins

Isolated IgG samples were dried in a vacuum centrifuge. After drying, IgG was denatured with the addition of 30 μl of 1.33% SDS (w/v) (Invitrogen, USA) and by incubation at 65 °C for 10 min. Plasma samples (10 μl) were denatured with the addition of 20 μl of 2% SDS (w/v) (Invitrogen, USA) and by incubation at 65 °C for 10 min. From this point on, the procedure was identical for both IgG and plasma samples. After denaturation, 10 μl of 4% Igepal-CA630 (v/v) (Sigma Aldrich, USA) was added to the samples, and the mixture was shaken 15 min on a plate shaker (GFL, Germany). N-glycans were released with the addition of 1.2 U of PNGase F (Promega, USA) and overnight incubation at 37 °C.

#### Fluorescent labelling and HILIC-SPE clean-up of released N-glycans

The released N-glycans were labelled with 2-aminobenzamide (2-AB). The labelling mixture consisted of 2-AB (19.2 mg/ml; Sigma Aldrich, USA) and 2-picoline borane (44.8 mg/ml; Sigma Aldrich, USA) in dimethyl sulfoxide (Sigma Aldrich, USA) and glacial acetic acid (Merck, Germany) mixture (70:30 v/v). To each sample, 25 μl of labelling mixture was added, followed by 2 h incubation at 65 °C. Free label and reducing agent were removed from the samples using hydrophilic interaction liquid chromatography solid-phase extraction (HILIC-SPE). After incubation samples were brought to 96% of acetonitrile (ACN) by adding 700 μl of ACN (J.T. Baker, USA) and applied to each well of a 0.2 μm GHP filter plate (Pall Corporation, USA). Solvent was removed by application of vacuum using a vacuum manifold (Millipore Corporation, USA). All wells were prewashed with 70% ethanol (Sigma-Aldrich, St. Louis, MO, USA) and water, followed by equilibration with 96% ACN. Loaded samples were subsequently washed 5× with 96% ACN. N-glycans were eluted with water and stored at −20 °C until usage.

#### Hydrophilic interaction liquid chromatography of N-glycans

Fluorescently labelled N-glycans were separated by hydrophilic interaction liquid chromatography (HILIC) on Acquity UPLC H-Class instrument (Waters, USA) consisting of a quaternary solvent manager, sample manager, and a fluorescence detector, set with excitation and emission wavelengths of 250 and 428 nm, respectively. The instrument was under the control of Empower 3 software, build 3471 (Waters, Milford, USA). Labelled N-glycans were separated on a Waters BEH Glycan chromatography column, with 100 mM ammonium formate, pH 4.4, as solvent A and ACN as solvent B. In the case of IgG N-glycans, separation method used linear gradient of 75–62% acetonitrile at flow rate of 0.4 ml/min in a 27-min analytical run. For plasma protein N-glycans separation method used linear gradient of 70–53% acetonitrile at flow rate of 0.561 ml/min in a 25-min analytical run. The system was calibrated using an external standard of hydrolysed and 2-AB labelled glucose oligomers from which the retention times for the individual glycans were converted to glucose units (GU). Data processing was performed using an automatic processing method with a traditional integration algorithm after which each chromatogram was manually corrected to maintain the same intervals of integration for all the samples. The chromatograms were all separated in the same manner into 24 peaks (GP1–GP24) for IgG N-glycans and 39 peaks (GP1–GP39) for plasma protein N-glycans and are depicted in Supplementary Fig. 2 and Supplementary Fig. 3, respectively. Detailed description of glycan structures corresponding to each glycan peak is presented in Supplementary Table 1. Glycan peaks were analysed based on their elution positions and measured in glucose units, then compared to the reference values in the “GlycoStore” database (available at: https://glycostore.org/) for structure assignment. The amount of glycans in each peak was expressed as a percentage of the total integrated area. For IgG N-glycans, in addition to 24 directly measured glycan traits, eight derived traits were calculated (Supplementary Table 2). In the case of TwinsUK cohort, IgG N-glycan derived traits were calculated from plasma protein glycan profiles, based on known elution positions of predominat IgG N-glycan structures (Supplementary Table 3). In general, derived glycan traits average particular glycosylation features, such as galactosylation, fucosylation, bisecting GlcNAc and sialylation.

### Statistical analysis

#### Bariatric cohort

In order to remove experimental variation from the measurements, normalisation and batch correction were performed on the UPLC glycan data. To make measurements across samples comparable, normalisation by total area was performed. Prior to batch correction, normalised glycan measurements were log-transformed due to right-skewness of their distributions and the multiplicative nature of batch effects. Batch correction was performed on log-transformed measurements using the ComBat method (R package sva) [31], where the technical source of variation (which sample was analysed on which plate) was modelled as batch covariate. To correct measurements for experimental noise, estimated batch effects were subtracted from log-transformed measurements.

Longitudinal analysis of patient samples through their observation period was performed by implementing a linear mixed effects model, where time was modelled as fixed effect, while the individual ID was modelled as random effect, without additional modelling of age. In regards to this, age was not included in the model since the follow-up period for Bariatric cohort was measured in months, therefore the changes in patients’ age are not relevant for glycosylation. Prior to the analyses, glycan variables were all transformed to standard normal distribution by inverse transformation of ranks to Normality (R package “GenABEL”, function rntransform). Using rank transformed variables makes estimated effects of different glycans comparable, as these will have the same standardised variance. False discovery rate (FDR) was controlled by the Benjamini–Hochberg procedure at the specified level of 0.05. Data were analysed and visualised using R programming language (version 3.5.2) [32].

#### TwinsUK cohort

Normalisation of peak intensities to the total chromatogram area was performed for each measured sample separately. Calculated proportions were then batch corrected using ComBat method (R package sva) [31]. Since only plasma N-glycoprofile data was available for the TwinsUK cohort, the extrapolation of the IgG N-glycoprofile from plasma N-glycoprofile had to be performed as this was the only way to deduce IgG N-glycosylation information from the available data. Previous studies demonstrated that neutral glycans in the total plasma protein N-glycoprofile originate nearly exclusively from immunoglobulins, mostly IgG [33], which allowed us to use the total plasma N-glycome data as a source for the IgG N-glycosylation. Mentioned neutral glycans which originate primarily from IgG are mostly located in the first 11 peaks of the total plasma N-glycome which were used to calculate six IgG derived glycan traits – agalactosylation (G0), monogalactosylation (G1), digalactosylation (G2), bisecting GlcNAc (B), core fucosylation (CF) and high mannose structures (HM). Prior to calculation of mentioned derived traits, the first 11 plasma glycan peaks had to be normalised to their total chromatogram area (calculated by adding up the areas under GP1, GP2, … GP11). For example, the relative abundance of GP1 was recalculated by dividing its area with the total IgG chromatogram area and multiplying with 100 (GP1/GP1 + GP2 + ⋯ + GP11 *100). Formulas used for the normalization of the first 11 plasma glycan peaks used for acquisition of IgG N-glycosylation data are presented in Supplementary Table 4. Mixed models were fitted to estimate the effect of BMI change on IgG N-glycome (R package lme4) [34]. Directly measured or derived glycan trait was used as a dependent variable in the mixed model. To differentiate between BMI change and the absolute BMI value, the variable was separated to BMI_baseline_ and BMI_difference_ (calculated according to the following equation: $BMI_{difference} = BMI_{follow\;up\;age}-BMI_{baseline\;age}$), and both were used in the model as a fixed effect. Since IgG N-glycome is affected by aging, and the follow-up period for the TwinsUK cohort was measured in years (average follow-up period ≈ 8 years) which resulted in significant change of participants’ age during the follow-up period, age was included both as a fixed effect and a random slope. Finally, to meet the independency criteria, family ID and individual ID (nested within family) were included in the model as a random intercept. Due to multiple model fitting (for 11 directly measured and six derived glycan traits) false discovery rate was controlled using Benjamini–Hochberg method. All statistical analyses were performed using R programming language (version 3.6.3) [32].

## Results

### Impact of pre-surgical low-calorie diet on IgG N-glycosylation

We chromatographically profiled the IgG N-glycome in a cohort of bariatric surgery-candidate patients before and after the pre-operative diet. By employing a linear mixed model, we observed significant change in only one out of eight examined IgG derived glycan traits. Namely, the levels of bisecting GlcNAc (B) were substantially decreased after the low-calorie diet intervention (Table 3), indicating a decreased proinflammatory potential of the circulating IgG. The other IgG N-glycosylation features did not exhibit significant alterations, possibly due to rather short follow-up period (3 weeks) and limited number of participants (*n* = 8) (Table 3). Graphical representation of the longitudinal alterations in IgG N-glycome after low-calorie diet are depicted in Supplementary Fig. 4.

**Table 3:** Impact of pre-surgical low-calorie diet on IgG glycosylation.

Derived IgG glycan trait	Time_effect	Time_SE	Time_*p* value	Adjusted *p* value
**Bisecting GlcNAc (B)**	−0.2801	0.0743	4.30 × 10^−03^	3.41 × 10^−02^
Agalactosylation (G0)	−0.0493	0.1876	7.93 × 10^−01^	7.93 × 10^−01^
Monogalactosylation (G1)	0.1122	0.1869	5.53 × 10^−01^	7.93 × 10^−01^
Digalactosylation (G2)	−0.0539	0.1387	6.99 × 10^−01^	7.93 × 10^−01^
Total sialylation (S)	0.0941	0.2090	6.54 × 10^−01^	7.93 × 10^−01^
Monosialylation (S1)	0.1354	0.1878	4.78 × 10^−01^	7.93 × 10^−01^
Disialylation (S2)	−0.0869	0.2474	7.27 × 10^−01^	7.93 × 10^−01^
Core fucosylation (CF)	0.0810	0.2416	7.39 × 10^−01^	7.93 × 10^−01^

Longitudinal analysis was performed by implementing a linear mixed effects model, with time as a fixed effect and the individual sample measurement as a random effect. False discovery rate was controlled using Benjamini–Hochberg method at the specified level of 0.05.

Bold – significant decrease; Underline – non-significant change.

*GlcNAc* N-acetylglucosamine, *SE* standard error.

### IgG N-glycosylation markedly changes after weight loss surgery

**Fig. 1:**
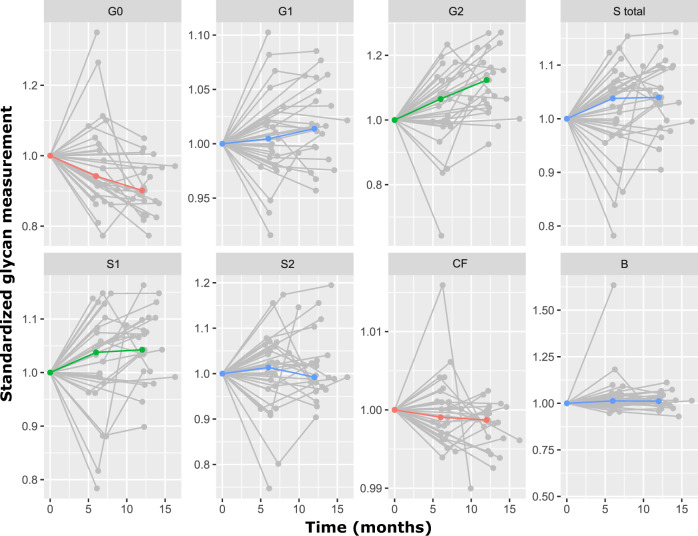
Bariatric surgery-related alterations in IgG N-glycosylation features over time (months). Standardised glycan measurements are represented on the *y-*axis, while time in months is presented on the *x-*axis. IgG N-glycosylation altered features: G0 – agalactosylation; G1 – monogalactosylation; G2 – digalactosylation; S total – total sialylation; S1 – monosialylation; S2 – disialylation; CF – core fucosylation; B – incidence of bisecting N-acetylglucosamine. Red line – significant decrease; green line – significant increase; blue line – non-significant change.

Using the same chromatographic approach, we analysed samples from patients who underwent bariatric surgery. The plasma samples were collected on the day of surgery (month 0), approximately 6 months post surgery and 12 months post surgery. IgG N-glycans were profiled in each of these timepoints, and the obtained values were used for derived glycan traits calculations. Statistical analysis revealed extensive changes in IgG N-glycome following the bariatric procedure. Namely, four out of eight tested derived traits showed marked changes: core fucosylated (CF) and agalactosylated (G0) glycans decreased, while digalactosylated (G2) and monosialylated (S1) glycans increased after the surgery (Table 4). The IgG N-glycans whose abundances were increased after bariatric surgery are major components of a young IgG N-glycome, as they are typically associated with a younger age. The opposite applies to agalactosylated structures, which are usual denominators of an old-like IgG N-glycome profile. We also examined the correlation of patients’ clinical data with IgG N-glycome features using multivariate analysis, but found no statistically significant associations (Supplementary Table 5). Finally, the type of bariatric surgery (either sleeve gastrectomy or Roux-en-Y gastric bypass) did not affect IgG N-glycome composition. Graphical representations of the longitudinal alterations in IgG N-glycosylation features are depicted in Fig. 1.

**Table 4:** Bariatric surgery induces significant changes in IgG N-glycome.

Derived IgG glycan trait	Time_effect	Time_SE	Time_*p* value	Adjusted *p* value
**Agalactosylation (G0)**	−0.0339	0.0078	9.23 × 10^−05^	7.38 × 10^−04^
Digalactosylation (G2)	0.0275	0.0072	3.75 × 10^−04^	1.50 × 10^−03^
Monosialylation (S1)	0.0193	0.0080	1.97 × 10^−02^	3.94 × 10^−02^
**Core fucosylation (CF)**	−0.0155	0.0064	1.74 × 10^−02^	3.94 × 10^−02^
*Total sialylation (S)*	0.0171	0.0083	4.70 × 10^−02^	6.27 × 10^−02^
*Bisecting GlcNAc (B)*	0.0173	0.0083	4.01 × 10^−02^	6.27 × 10^−02^
*Monogalactosylation (G1)*	0.0206	0.0107	8.18 × 10^−02^	9.35 × 10^−02^
*Disialylation (S2)*	0.0048	0.0080	5.48 × 10^−01^	5.48 × 10^−01^

Longitudinal analysis was performed by implementing a linear mixed effects model, with time as a fixed effect and the individual sample measurement as a random effect. False discovery rate was controlled using Benjamini–Hochberg method at the specified level of 0.05.

Bold – significant decrease; Underline – significant increase; Italic – non-significant change.

*GlcNAc* N-acetylglucosamine, *SE* standard error.

### Weight loss induces a shift towards young-like IgG N-glycome

**Fig. 2:**
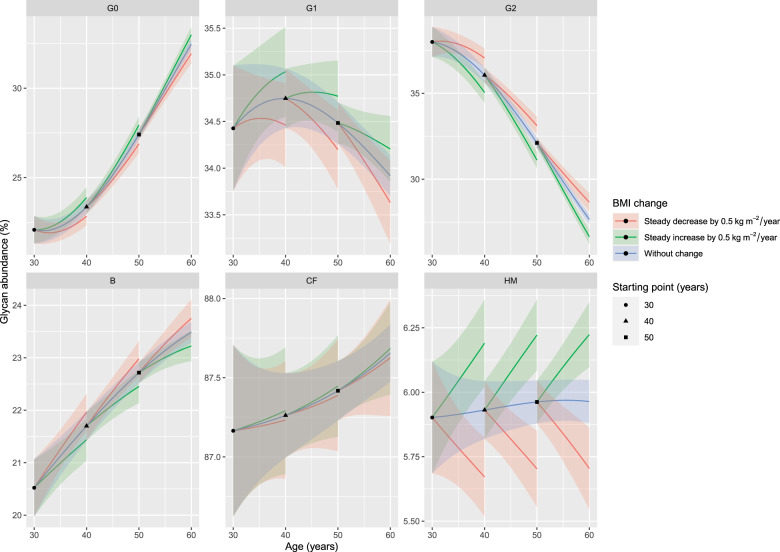
BMI-associated alterations in IgG N-glycosylation across multiple timepoints. Changes in IgG N-glycosylation derived traits are presented with lineplots of hypothetical ageing of TwinsUK participants (all women). Black dot represents a starting point of a 30-year-old woman, black triangle of a 40-year-old woman and black square of a 50-year-old woman. All of these women have a baseline BMI of 25 kg/m^2^. Blue lines represent age-related IgG N-glycosylation changes attributed to stabile BMI. Green lines represent age-related IgG N-glycosylation changes attributed to increasing BMI (0.5 kg/m^2^ per year, through a period of 10 years). Red lines represent age-related IgG N-glycosylation changes attributed to decreasing BMI (0.5 kg/m^2^ per year, through a period of 10 years). Highlighted areas represent 95% confidence intervals. The effect of age on IgG N-glycosylation is represented with the curve slope, while the effect of BMI change is represented with the distance of green/red line from the blue line.

Using the same chromatographic approach, we profiled the plasma protein N-glycome from 1680 TwinsUK study participants sampled at several timepoints over a 20-year-period. This served as a replication of the findings from the bariatric cohort, whose participants exhibited the reversal from old- to young-like IgG N-glycome due to weight loss. Due to the fact that for the TwinsUK cohort only plasma N-glycome was profiled and therefore available, we calculated derived traits and performed statistical analysis based only on the first 11 plasma glycan peaks, corresponding to the glycans originating nearly exclusively from IgG as demonstrated in previous studies [33]. We examined IgG N-glycome alterations associated with changes in BMI using a mixed model on a subset of 3742 samples. Out of six examined IgG N-glycosylation features (derived traits), three displayed significant BMI-decrease-related changes – agalactosylation (G0), digalactosylation (G2) and incidence of high mannose structures (HM) (Table 5). Namely, the abundance of digalactosylated (G2) glycans increased with the BMI decrease, while the abundance of agalactosylated (G0) and high mannose glycans (HM) decreased with the weight loss, estimated by the BMI drop. These findings are in line with the results observed in the bariatric surgery cohort. Graphical representation of the longitudinal BMI-dependent alterations of IgG N-glycosylation is depicted in Fig. 2.

**Table 5:** Longitudinally monitored weight loss-associated significant changes of IgG N-glycosylation.

Derived IgG glycan trait	BMI difference effect (glycan abundance (%) change per 1 kg/m^2^ decrease in BMI)	BMI difference SE (glycan abundance (%) change per 1 kg/m^2^ decrease in BMI)	*p* value	Adjusted *p* value
Digalactosylation (G2)	0.2004	0.0403	6.88 × 10^−07^	5.85 × 10^−06^
**High mannose (HM)**	−0.0519	0.0119	1.33 × 10^−05^	5.66 × 10^−05^
**Agalactosylation (G0)**	−0.1048	0.0397	8.43 × 10^−03^	1.79 × 10^−02^
*Bisecting GlcNAc (B)*	0.0526	0.0262	4.49 × 10^−02^	6.94 × 10^−02^
*Monogalactosylation (G1)*	−0.0573	0.0343	9.53 × 10^−02^	1.25 × 10^−01^
*Core fucosylation (CF)*	−0.0059	0.0285	8.36 × 10^−01^	8.89 × 10^−01^

Longitudinal analysis was performed by implementing a mixed model, fitted to estimate the effect of BMI change on IgG N-glycome. False discovery rate was controlled using Benjamini–Hochberg method at the specified level of 0.05.

Bold – significant decrease; Underline – significant increase; Italic – non-significant change.

*BMI* body mass index, *GlcNAc* *N*-acetylglucosamine, *SE* standard error.

## Discussion

In this study, we have observed extensive changes in the IgG N-glycome associated with weight loss following a low-calorie diet, bariatric surgery, or a decrease of BMI with time. To the best of our knowledge, this is the first study to investigate IgG N-glycome alterations in patients who underwent a low-calorie diet followed by bariatric surgery.

Prior to bariatric surgery, patients were subjected to a 3-week low-calorie diet which induced a single significant change to the IgG N-glycome in the form of reduced levels of bisecting GlcNAc after the dieting period. In general, higher levels of bisecting GlcNAc are associated with enhanced affinity for FcγRs and, consequently, with enhanced antibody-dependent cellular cytotoxicity (ADCC) and other effector functions of the immune cells [9]. Hence, the reduction of bisecting GlcNAc levels on IgG decreases IgG inflammatory potential. Furthermore, several studies have reported a sex-independent increase of bisecting GlcNAc levels with age [35, 36], suggesting that this diet-related decrease also contributes to the reduction of the biological age. Increased abundance of bisecting GlcNAc has also been previously associated with type 2 diabetes [37] and with higher cardiovascular risk [13], therefore implying that dieting improves individual’s cardiometabolic health through altered IgG glycosylation.

We have also analysed IgG N-glycome from individuals who underwent bariatric surgery, in a longitudinal manner. We observed several significant changes in IgG N-glycome, such as a marked decrease in agalactosylated (G0) IgG. Elevated levels of G0 IgG glycoforms are typically associated with ageing, pro-inflammatory IgG glycan profile and various inflammatory diseases [9]. On the other hand, the levels of digalactosylated (G2) glycans increased after bariatric surgery and at sequential timepoints, in accordance with a reduced inflammatory potential of the circulating IgG. The increased levels of IgG galactosylation were previously associated with younger biological age and are considered, in a way, as a measure of an individual’s well-being [9, 12]. Our results demonstrate that weight loss, resulting from bariatric surgery, can initiate the reversal from an old-like to a young-like IgG N-glycome, potentially reversing the clock for biological age. Bariatric surgery-related weight loss also led to an increase in IgG sialylation, which is the main modulator of the IgG anti-inflammatory actions [38]. In addition to its anti-inflammatory actions, the level of IgG sialylation has been implicated in the pathogenesis of obesity-induced insulin resistance and hypertension, as already mentioned [19, 20]. It was shown that hyposialylated IgG acts as an operating ligand of inhibitory IgG receptor FcγRIIB, found to be expressed in the microvascular endothelium, leading to the induction of obesity-related insulin resistance and hypertension. On the contrary, the sialylated glycoform is preserving insulin sensitivity and normal vasomotor tone, even in obese mice. Interestingly, the same group made another significant discovery – supplementation with sialic acid precursor restores IgG sialylation, highlighting a potential approach to improve both metabolic and cardiovascular health in humans, with a single intervention [20, 24]. Our data suggest that a similar effect might be achieved by weight loss interventions. Lastly, bariatric surgery also resulted in a significant decrease in core fucosylation (CF), a glycosylation feature present on the vast majority of circulating IgG molecules (approximately 95%). Although we have noticed a strong anti-inflammatory pattern of the changes in IgG N-glycome, this decrease in core fucosylation is associated with an increase in IgG binding affinity to FcɣRIIIA receptor and sequential ADCC [39].

In order to confirm the effects of weight loss on biological age, we investigated how a decreasing BMI affects the IgG N-glycome during a 20-year-period. We observed the prominent inverse changes of agalactosylated (G0) and digalactosylated (G2) IgG N-glycans––agalactosylated IgG N-glycans significantly decreased, while digalactosylated ones substantially increased as the BMI decreased. These observations corroborated our findings from the bariatric patients, confirming that the body weight reduction reverses IgG N-glycome from old-like to young-like, implying at the same time a likely reduction in the biological age. There are several limitations to our study. First, only eight participants had their blood drawn prior to the low-calorie diet which had a relatively short follow-up time (3 weeks). Despite the reduced sample size, the diet data on the obese patients show a remarkable effect of caloric restriction on IgG N-glycosylation. In fact, while IgG N-glycans repertoire remains quite stable under homeostatic conditions, the extent of variation triggered by a calorie-restricted regimen shows the possibility of modulating the ageing process through metabolic intervention. Second, TwinsUK participants have not experienced such an extensive weight loss, which potentially influenced the replication of other significant glycan changes from the bariatric cohort. Third, the weight loss in TwinsUK cohort was approximated by BMI decrease, which is usually a legitimate assumption, however, it does not have to apply to all cases. Finally, we profiled plasma N-glycome in the TwinsUK cohort, while the IgG glycan traits were approximated and the information on IgG sialylation was confounded by other plasma glycoproteins. Ideally, these issues could be circumvented in future studies with an experimental design that would allow simultaneous, multi-centre follow-up of larger groups of patients.

## Conclusion

To summarise, our results indicate that both dieting and bariatric surgery have an impact on inflammation and biological ageing by altering IgG N-glycan patterns. All of the observed weight-loss-associated alterations in IgG N-glycosylation are suggesting a decreased inflammatory potential of the circulating IgG and a reduction of biological age.

## Supplementary information

Supplementary material

## Data Availability

All results presented in this paper were generated using R programming language as mentioned previously. Code used for the assessment of the results can be available upon request to the authors.
