# Supplementary material for: Extensive weight loss reduces glycan age by altering IgG N-glycosylation
Source: Int J Obes (Lond). 2021 May 3;45(7):1521–31. doi: 10.1038/s41366-021-00816-3 (PMC8236401; doi:10.1038/s41366-021-00816-3)
Supplement: Supplementary file 1 — Supplementary material [file 41366_2021_816_MOESM1_ESM.pdf]

Supplementary files

Extensive weight loss reduces glycan age by altering IgG N-glycosylation

Valentina L Greto[1]*, Ana Cvetko[2]*, Tamara Štambuk[2,3]*, Niall J Dempster[4], Domagoj Kifer[2], Helena Deriš[3], Ana Cindrić[3], Frano Vučković[3], Mario Falchi[5], Richard S Gillies[6], Jeremy W Tomlinson[4], Olga Gornik[2,3], Bruno Sgromo[6], Tim D Spector[5], Cristina Menni[5]*, Alessandra Geremia[1]*, Carolina V Arancibia- Cárcamo[1]*, Gordan Lauc[2,3]*

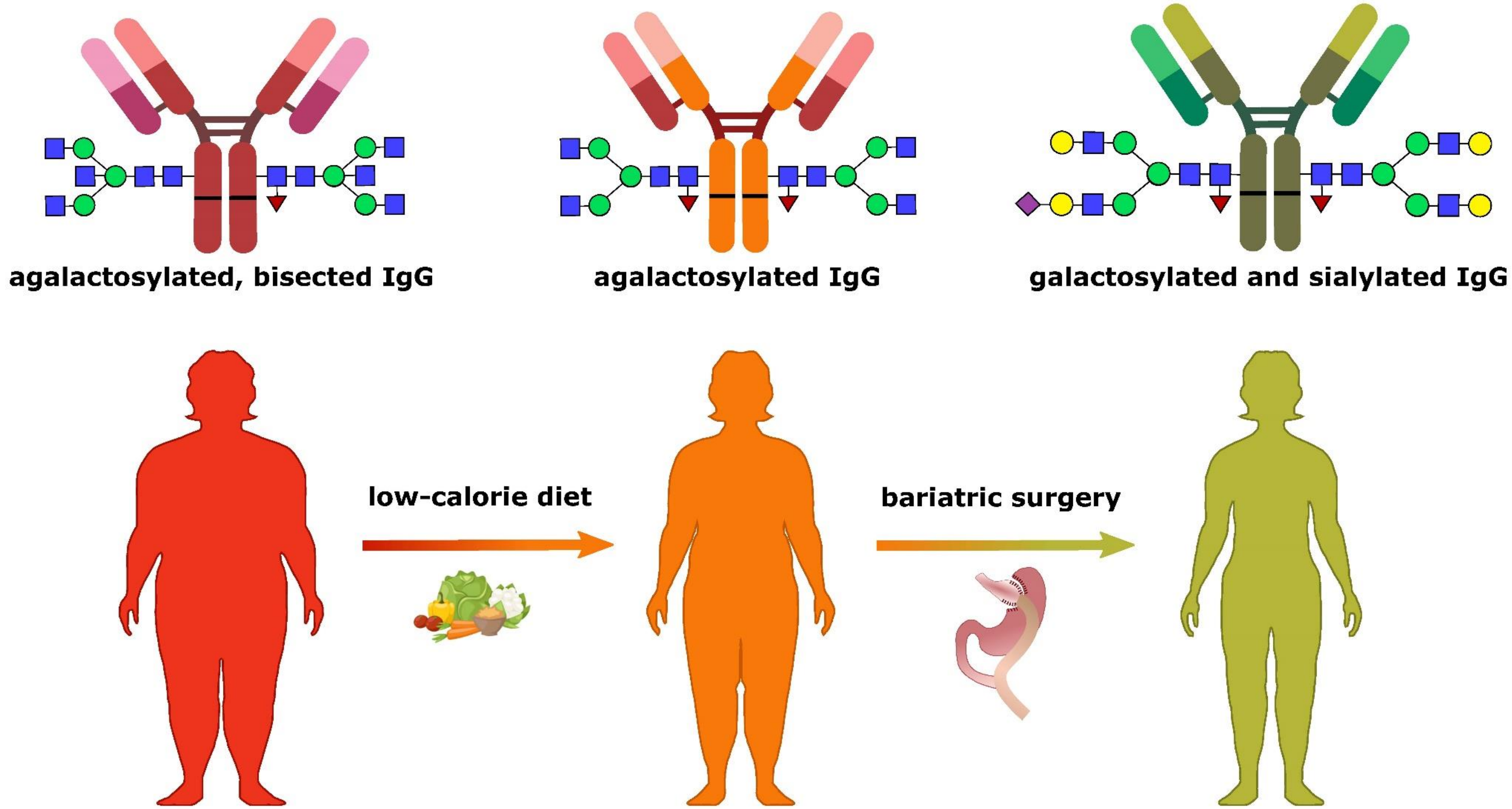

Supplementary Figure 1 Graphical abstract (cover art).

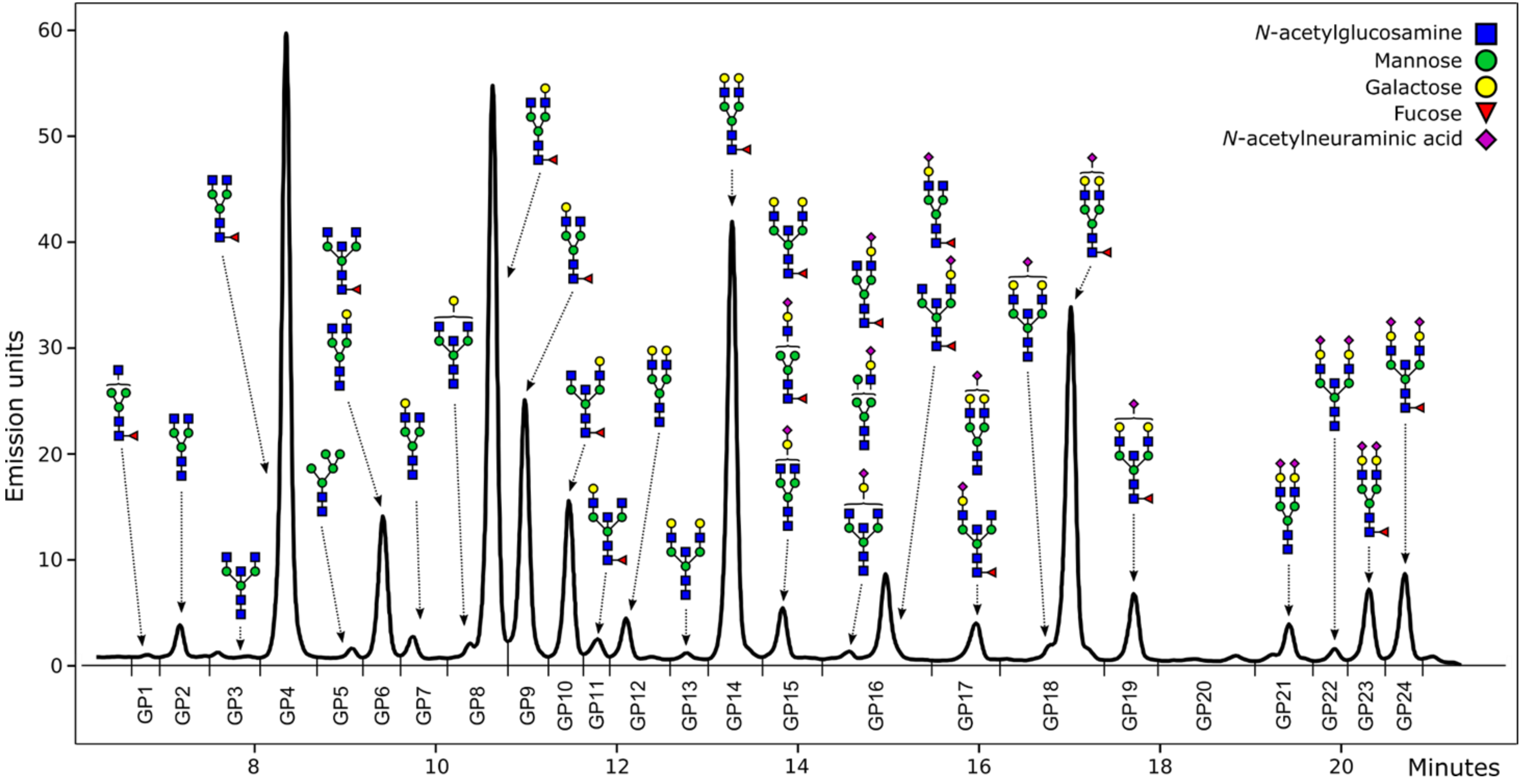

Supplementary Figure 2 Representative HILIC-UPLC-FLR chromatographic profile of IgG N-glycome, with graphic representation of the glycan structures corresponding to each glycan peak (GP).

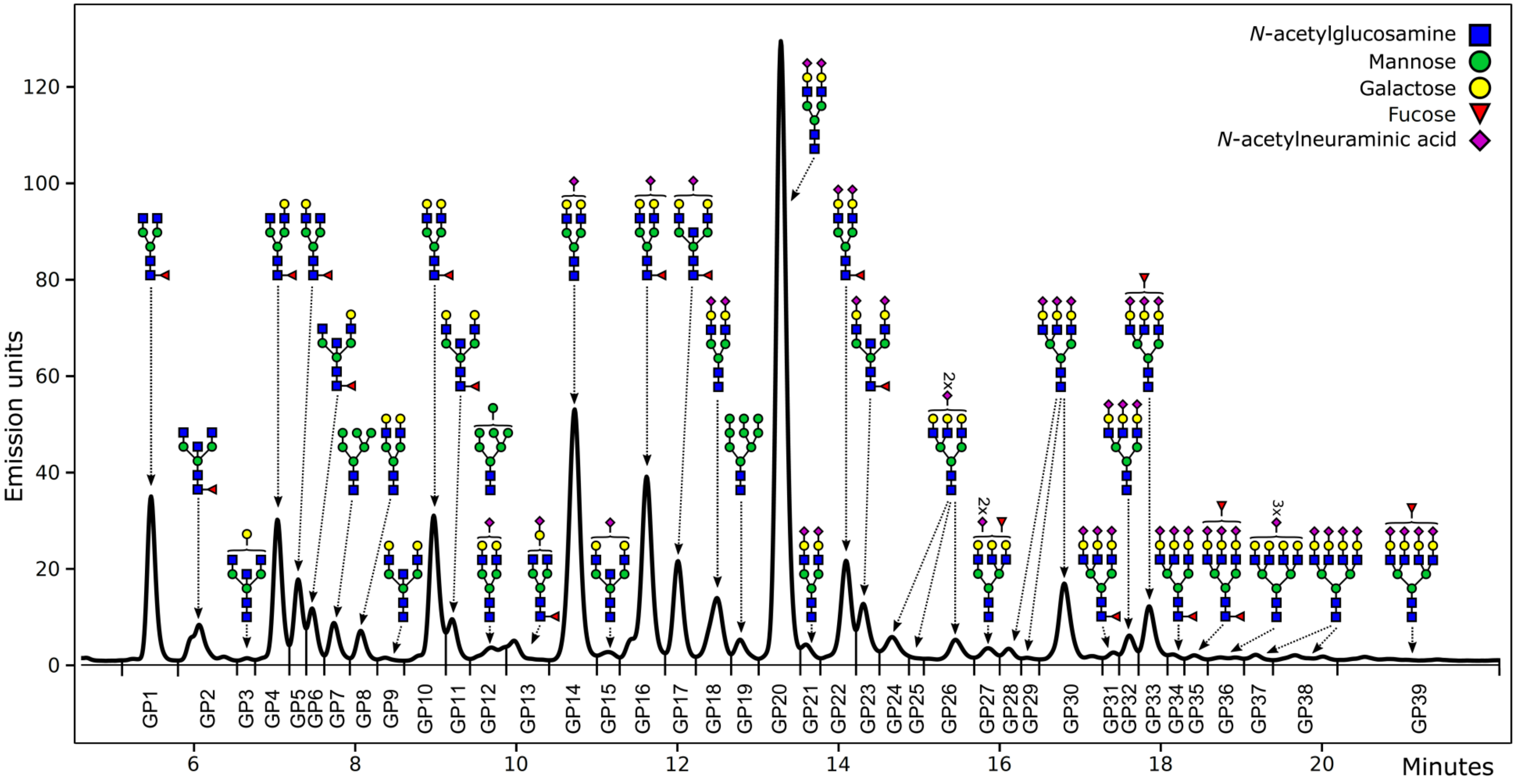

Supplementary Figure 3 Representative HILIC-UPLC-FLR chromatographic profile of plasma N-glycome, with graphic representation of the most abundant glycan structure corresponding to each glycan peak (GP).

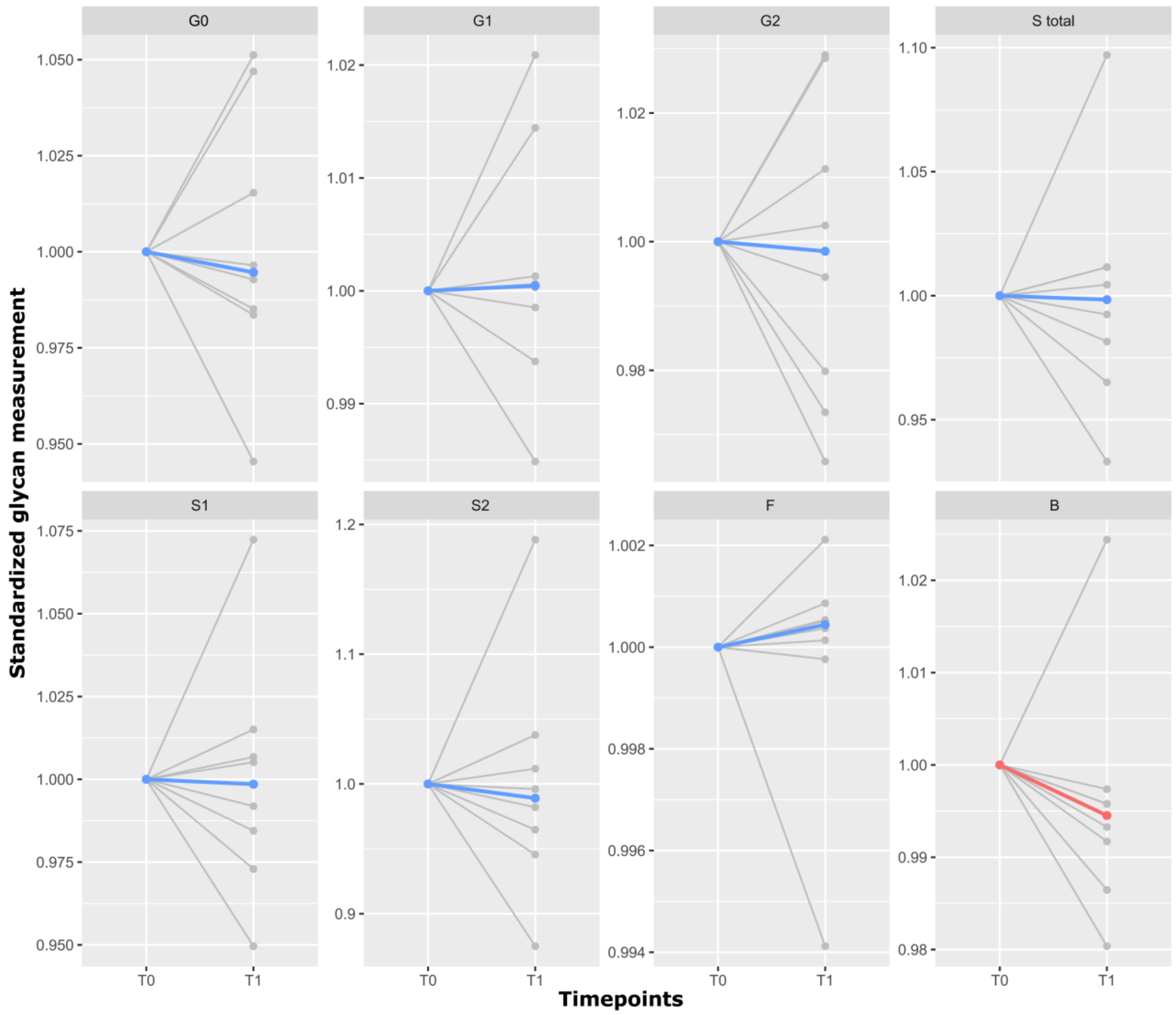

Supplementary Figure 4 Low-calorie diet-associated alterations in IgG N-glycosylation features after a 3-week follow-up. Standardized glycan measurements are represented on the y axis, while time in months is presented on the x axis. IgG N-glycosylation altered features: G0 – agalactosylation; G1 – monogalactosylation; G2 – digalactosylation; S total – total sialylation; S1 – monosialylation; S2 – disialylation; F – fucosylation; B – incidence of bisecting N-acetylglucosamine. Red line – significant decrease; green line – significant increase; blue line – non-significant change.

Supplementary Table 1 Detailed description of glycan structures corresponding to every individual IgG and plasma glycan peak.*

Glycome	*Glycan peak*	*Glycan structure*	*Description*	*Formula*
IgG	GP1	FA1	core fucosylated, monoantennary	GP1 / GP * 100
IgG	GP2	A2	agalactosylated, biantennary	GP2 / GP * 100
IgG	GP3	A2B	biantennary with bisecting GlcNAc	GP3 / GP * 100
IgG	GP4	FA2	core fucosylated, biantennary	GP4 / GP * 100
IgG	GP5	M5	oligomannose	GP5 / GP * 100
IgG	GP6	FA2B;<br>A2[6]G1	core fucosylated, biantennary with bisecting GlcNAc;<br>monogalactosylated, biantennary	GP6 / GP * 100
IgG	GP7	A2[3]G1	monogalactosylated, biantennary	GP7 / GP * 100
IgG	GP8	A2BG1;<br>FA2[6]G1	monogalactosylated, biantennary with bisecting GlcNAc;<br>core fucosylated and monogalactosylated, biantennary	GP8 / GP * 100
IgG	GP9	FA2[3]G1	core fucosylated and monogalactosylated, biantennary	GP9 / GP * 100
IgG	GP10	FA2[6]BG1	core fucosylated and monogalactosylated, biantennary with bisecting GlcNAc	GP10 / GP * 100
IgG	GP11	FA2[3]BG1	core fucosylated and monogalactosylated, biantennary with bisecting GlcNAc	GP11 / GP * 100
IgG	GP12	A2G2	digalactosylated, biantennary	GP12 / GP * 100
IgG	GP13	A2BG2	digalactosylated, biantennary with bisecting GlcNAc	GP13 / GP * 100
IgG	GP14	FA2G2	core fucosylated, digalactosylated, biantennary	GP14 / GP * 100
IgG	GP15	A2G1S1;<br>FA1G1S1;<br>FA2BG2	monogalactosylated and monosialylated biantennary;<br>core fucosylated, monogalactosylated and monosialylated monoantennary; core fucosylated, digalactosylated, biantennary with bisecting GlcNAc	GP15 / GP * 100
IgG	GP16	A2BG1S1;<br>FA2[6]G1S1;<br>M1A1G1S1;<br>FA2[6]BG1S1;<br>FA2[3]G1S1	monogalactosylated and monosialylated biantennary with bisecting GlcNAc;<br>core fucosylated, monogalactosylated and monosialylated biantennary;<br>monomannosylated, monogalactosylated and monosialylated biantennary;<br>core fucosylated, monogalactosylated, monosialylated biantennary with bisecting GlcNAc;<br>core fucosylated, monogalactosylated and monosialylated biantennary	GP16 / GP * 100
IgG	GP17	FA2[3]BG1S1;<br>A2G2S1	core fucosylated, monogalactosylated, monosialylated biantennary with bisecting GlcNAc;<br>digalactosylated and monosialylated biantennary	GP17/ GP * 100
IgG	GP18	A2BG2S1;	digalactosylated and monosialylated biantennary with bisecting GlcNAc;	GP18 / GP * 100

		FA2G2S1	core fucosylated, digalactosylated and monosialylated biantennary	
IgG	GP19	FA2BG2S1	core fucosylated, digalactosylated and monosialylated biantennary with bisecting GlcNAc	GP19 / GP * 100
IgG	GP20	structure not determined		GP20 / GP * 100
IgG	GP21	A2G2S2	digalactosylated and disialylated biantennary	GP21 / GP * 100
IgG	GP22	A2BG2S2	digalactosylated and disialylated biantennary with bisecting GlcNAc	GP22 / GP * 100
IgG	GP23	FA2G2S2	core fucosylated, digalactosylated and disialylated biantennary	GP23 / GP * 100
IgG	GP24	FA2BG2S2	core fucosylated, digalactosylated and disialylated biantennary with bisecting GlcNAc	GP24 / GP * 100
Glycome	***Glycan peak***	***Glycan structure***	***Description***	***Formula***
plasma	GP1	FA2	core fucosylated, biantennary	GP1 / GP * 100
plasma	GP2	FA2B; M5	core fucosylated, biantennary with bisecting GlcNAc;<br>oligomannose	GP2 / GP * 100
plasma	GP3	A2BG1	monogalactosylated, biantennary with bisecting GlcNAc	GP3 / GP * 100
plasma	GP4	FA2[6]G1	core fucosylated and monogalactosylated, biantennary	GP4 / GP * 100
plasma	GP5	FA2[3]G1	core fucosylated and monogalactosylated, biantennary	GP5 / GP * 100
plasma	GP6	FA2[6]BG1	core fucosylated and monogalactosylated, biantennary with bisecting GlcNAc	GP6 / GP * 100
plasma	GP7	M6;<br>FA2[3]BG1	oligomannose;<br>core fucosylated and monogalactosylated, biantennary with bisecting GlcNAc	GP7 / GP * 100
plasma	GP8	A2G2	digalactosylated, biantennary	GP8 / GP * 100
plasma	GP9	A2BG2	digalactosylated, biantennary with bisecting GlcNAc	GP9 / GP * 100
plasma	GP10	FA2G2	core fucosylated, digalactosylated, biantennary	GP10 / GP * 100
plasma	GP11	FA2BG2	core fucosylated, digalactosylated, biantennary with bisecting GlcNAc	GP11 / GP * 100
plasma	GP12	M7;<br>A2G2S1;<br>A1M4G1S1;<br>A2BG1S1	oligomannose;<br>digalactosylated, monosialylated, biantennary;<br>tetramannosylated, monogalactosylated, monosialylated, monoantennary;<br>monogalactosylated, monosialylated, biantennary with bisecting GlcNAc	GP12 / GP * 100
plasma	GP13	FA2G1S1;<br>FA2BG1S1	core fucosylated, monogalactosylated and monosialylated biantennary;<br>core fucosylated, monogalactosylated and monosialylated biantennary with bisecting GlcNAc	GP13 / GP * 100

plasma	GP14	A2G2S1	digalactosylated and monosialylated biantennary	GP14 / GP * 100
plasma	GP15	A2BG2S1	digalactosylated and monosialylated biantennary with bisecting GlcNAc	GP15 / GP * 100
plasma	GP16	FA2G2S1	core fucosylated, digalactosylated and monosialylated biantennary	GP16 / GP * 100
plasma	GP17	FA2BG2S1	core fucosylated, digalactosylated and monosialylated biantennary with bisecting GlcNAc	GP17/ GP * 100
plasma	GP18	A2G2S2;<br>FA2G2S2	digalactosylated and disialylated biantennary;<br>core fucosylated, digalactosylated and disialylated biantennary	GP18 / GP * 100
plasma	GP19	M9	oligomannose	GP19 / GP * 100
plasma	GP20	A2G2S2	digalactosylated and disialylated biantennary	GP20 / GP * 100
plasma	GP21	A2G2S2;<br>A3G3S1;<br>FA2G2S2;<br>A2BG2S2;<br>A3F1G3S1	digalactosylated and disialylated biantennary;<br>trigalactosylated and monosialylated triantennary;<br>core fucosylated, digalactosylated and disialylated biantennary;<br>digalactosylated and disialylated biantennary with bisecting GlcNAc;<br>antennary fucosylated, trigalactosylated and monosialylated triantennary	GP21 / GP * 100
plasma	GP22	FA2G2S2	core fucosylated, digalactosylated and disialylated biantennary	GP22 / GP * 100
plasma	GP23	FA2BG2S2	core fucosylated, digalactosylated and disialylated biantennary with bisecting GlcNAc	GP23 / GP * 100
plasma	GP24	A3G3S2;<br>A3F1G3S1	trigalactosylated and disialylated triantennary;<br>antennary fucosylated, trigalactosylated and monosialylated triantennary	GP24 / GP * 100
plasma	GP25	A3G3S2;<br>FA2F1G2S2;<br>A3F1G3S2	trigalactosylated and disialylated triantennary;<br>core fucosylated, antennary fucosylated, digalactosylated and disialylated biantennary;<br>antennary fucosylated, trigalactosylated and disialylated triantennary	GP25 / GP * 100
plasma	GP26	A3G3S2;<br>FA3G3S2	trigalactosylated and disialylated triantennary;<br>core fucosylated, trigalactosylated and disialylated triantennary	GP26 / GP * 100
plasma	GP27	A3F1G3S2;<br>A3G3S3	antennary fucosylated, trigalactosylated and disialylated triantennary;<br>trigalactosylated and trisialylated triantennary	GP27 / GP * 100
plasma	GP28	A3G3S3;<br>A3F1G3S2	trigalactosylated and trisialylated triantennary;<br>antennary fucosylated, trigalactosylated and disialylated triantennary	GP28 / GP * 100
plasma	GP29	A3G3S3;<br>A3F1G3S2	trigalactosylated and trisialylated triantennary;<br>antennary fucosylated, trigalactosylated and disialylated triantennary	GP29 / GP * 100
plasma	GP30	A3G3S3;<br>A3F1G3S3	trigalactosylated and trisialylated triantennary;<br>antennary fucosylated, trigalactosylated and trisialylated triantennary	GP30 / GP * 100
plasma	GP31	FA3G3S3;	core fucosylated, trigalactosylated and trisialylated triantennary;	GP31 / GP * 100

		A3G3S3	trigalactosylated and trisialylated triantennary	
plasma	GP32	A3G3S3	trigalactosylated and trisialylated triantennary	GP32 / GP * 100
plasma	GP33	A3F1G3S3	antennary fucosylated, trigalactosylated and trisialylated triantennary	GP33 / GP * 100
plasma	GP34	FA3G3S3;<br>A4G4S3	core fucosylated, trigalactosylated and trisialylated triantennary;<br>tetragalactosylated and trisialylated tetraantennary	GP34 / GP * 100
plasma	GP35	FA3F1G3S3;<br>A4F1G4S3	core fucosylated, antennary fucosylated, trigalactosylated and trisialylated triantennary;<br>antennary fucosylated, tetragalactosylated and trisialylated tetraantennary	GP35 / GP * 100
plasma	GP36	A4G4S3;<br>A4F1G4S3	tetragalactosylated and trisialylated tetraantennary;<br>antennary fucosylated, tetragalactosylated and trisialylated tetraantennary	GP36 / GP * 100
plasma	GP37	A4G4S4;<br>A4F1G4S3	tetragalactosylated and tetrasialylated tetraantennary;<br>antennary fucosylated, tetragalactosylated and trisialylated tetraantennary	GP37 / GP * 100
plasma	GP38	A4G4S4;<br>A4F1G4S4;<br>A4F1G4S3	tetragalactosylated and tetrasialylated tetraantennary;<br>antennary fucosylated, tetragalactosylated and tetrasialylated tetraantennary;<br>antennary fucosylated, tetragalactosylated and trisialylated tetraantennary	GP38 / GP * 100
plasma	GP39	A4F1G4S4;<br>A4F2G4S4	antennary fucosylated, tetragalactosylated and tetrasialylated tetraantennary;<br>antennary difucosylated, tetragalactosylated and tetrasialylated tetraantennary;	GP39 / GP * 100

*structure abbreviations – all N-glycans have two core *N*-acetylglucosamines (GlcNAcs); F at the start of the abbreviation indicates a core-fucose α1,6-linked to the inner GlcNAc; Mx, number (x) of mannose on core GlcNAcs; Ax, number of antenna (GlcNAc) on trimannosyl core; A2, biantennary with both GlcNAcs as β1,2-linked; A3, triantennary with a GlcNAc linked β1,2 to both mannose and the third GlcNAc linked β1,4 to the α1,3 linked mannose; A4, GlcNAcs linked as A3 with additional GlcNAc β1,6 linked to α1,6 mannose; B, bisecting GlcNAc linked β1,4 to β1,3 mannose; G(x), number (x) of β1,4 linked galactose on antenna; F(x), number (x) of fucose linked α1,3 to antenna GlcNAc; S(x), number (x) of sialic acids linked to galactose

Supplementary Table 2 Immunoglobulin G derived glycan traits calculated out of 24 directly measured IgG glycan peaks in the Bariatric cohort.

Structural feature	***Formula***
Agalactosylation (G0)	GP1+GP2+GP4+GP6
Monogalactosylation (G1)	GP7+GP8+GP9+GP10+GP11+GP16
Digalactosylation (G2)	GP12+GP13+GP14+GP15+GP17+GP18+GP19+GP21+GP22+GP23+GP24
Total sialylation (S)	GP16+GP17+GP18+GP19+ GP21+GP22+GP23+GP24
Monosialylation (S1)	GP16+GP17+GP18+GP19
Disialylation (S2)	GP21+GP22+GP23+GP24
Incidence of bisecting GlcNAc (B)	GP6+GP10+GP11+GP13+GP15+GP19+GP22+GP24
Core fucosylation (CF)	GP1+GP4+GP6+GP8+GP9+GP10+GP11+GP14+GP15+GP16+GP18+GP19+GP23+GP24

Supplementary Table 3 Immunoglobulin G derived glycan traits calculated out of 11 directly measured plasma glycan peaks, corresponding to glycan structures which predominantly originate from IgG in the TwinsUK cohort.

Structural feature	***Formula***
Agalactosylation (G0)	GP1 + GP2
Monogalactosylation (G1)	GP3 + GP4 + GP5 + GP6
Digalactosylation (G2)	GP8 + GP9 + GP10 + GP11
Incidence of bisecting GlcNAc (B)	GP2 + GP3 + GP6 + GP9 + GP11
Core fucosylation (CF)	GP1 + GP2 + GP4 + GP5 + GP6 + GP10 + GP11
High mannose glycans (HM)	GP7

Supplementary Table 4 Formulas used for the for the recalculation of the relative abundances of the first 11 glycan peaks from the total plasma N-glycome of the TwinsUK cohort for the acquisition of IgG N-glycoprofile from the plasma N-glycome data.

Glycan peak	***Formula***
GP1	GP1/GP1 + GP2 + … + GP11 *100
GP2	GP2/GP1 + GP2 + … + GP11 *100
GP3	GP3/GP1 + GP2 + … + GP11 *100
GP4	GP4/GP1 + GP2 + … + GP11 *100
GP5	GP5/GP1 + GP2 + … + GP11 *100
GP6	GP6/GP1 + GP2 + … + GP11 *100
GP7	GP7/GP1 + GP2 + … + GP11 *100
GP8	GP8/GP1 + GP2 + … + GP11 *100
GP9	GP9/GP1 + GP2 + … + GP11 *100
GP10	GP10/GP1 + GP2 + … + GP11 *100
GP11	GP11/GP1 + GP2 + … + GP11 *100

Supplementary Table 5 Correlation of Bariatric cohort patients' clinical data with IgG N-glycome features.

Derived IgG glycan trait	***Clinical trait***	***Time_effect***	***Time_SE***	***Time_p-value***	***Adjusted p-value***
G1 total	CholHdlRatio	1.6852	0.5823	0.007	0.163
B total	HbA1c	-1.2644	0.7466	0.102	0.997
F total	HbA1c	0.911	0.5951	0.131	0.997
G2 total	CholHdlRatio	-0.388	0.3926	0.325	0.997
G1 total	BMI	0.9896	0.9993	0.343	0.997
G2 total	AST	-0,1898	0.2051	0.364	0.997
G2 total	BMI	0.6077	0.6617	0.369	0.997
B total	CholHdlRatio	0.4106	0.5334	0.445	0.997
B total	BMI	-0.5787	0.7853	0.468	0.997
G0 total	AST	0.127	0.2124	0.555	0.997
S total	AST	-0.1325	0.2261	0.561	0.997
G1 total	AST	0.1717	0.2926	0.563	0.997
F total	AST	-0.0845	0,1812	0.643	0.997
F total	CholHdlRatio	-0.1706	0.3614	0.645	0.997
G2 total	HbA1c	0.2108	0.6387	0.743	0.997
G0 total	BMI	-0.2254	0.6915	0.748	0.997
F total	BMI	0.1208	0.5992	0.85	0.997
G0 total	CholHdlRatio	-0.0614	0,4096	0.881	0.997
S total	HbA1c	-0.0941	0.7505	0.9	0.997
G0 total	HbA1c	-0.0706	0.6736	0.916	0.997
B total	AST	0.0181	0.2413	0.94	0.997
S total	BMI	-0.0372	0.7361	0.96	0.997
S total	CholHdlRatio	0.0081	0.4752	0.987	0.997
G1 total	HbA1c	0.0037	0.9894	0.997	0.997

Clinical traits` abbreviations - total cholesterol to HDL ratio (CholHdlRatio); glycated haemoglobin (HbA1c); Body Mass Index (BMI); Aspartate Aminotransferase (AST)
